# Supplementary material for: Angiopoietin-2–integrin α5β1 signaling enhances vascular fatty acid transport and prevents ectopic lipid-induced insulin resistance
Source: Nat Commun. 2020 Jun 12;11:2980. doi: 10.1038/s41467-020-16795-4 (PMC7293240; doi:10.1038/s41467-020-16795-4)
Supplement: Supplementary file 1 — Supplementary Information [file 41467_2020_16795_MOESM1_ESM.pdf]

Supplementary Information

Angiopoietin-2–integrin α5β1 signaling enhances vascular fatty acid transport and prevents ectopic lipid–induced insulin resistance

Bae *et al.*

It includes;

1. Supplementary Figures 1-13
2. Supplementary Tables 1-3

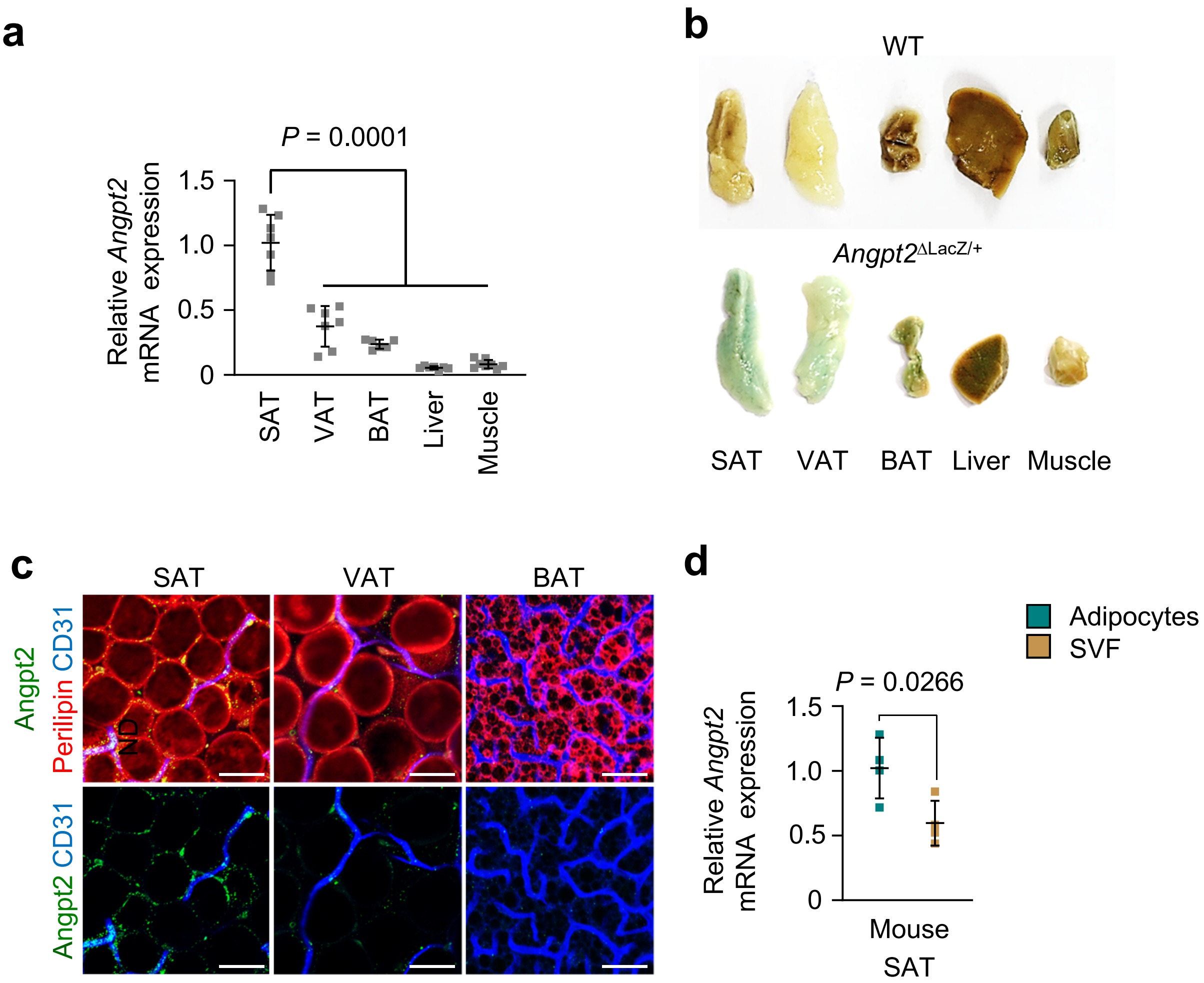

Supplemental Fig. 1 Angpt2 is preferentially expressed in adipocytes of SAT among metabolic organs

a Comparison of Angpt2 mRNA expression in different adipose tissues and metabolic organs. Each dot indicates a value obtained from one mouse and *n* = 6 (BAT), 7 (SAT,BAT, and Liver), 8 (Muscle) mice/group pooled from two independent experiments. Horizontal bars indicate mean ± SD and *P* values versus SAT by by one-way ANOVA. **b** Representative images of darkish-green β-galactosidase activity reflecting Angpt2 expression in different adipose tissues and metabolic organs of WT (negative control) and *Angpt2*$^{\Delta LacZ/+}$ reporter mice. **c** Representative images of immunofluorescence staining of Angpt2 in different adipose tissues of *Angpt2*-eGFP reporter mice. Scale bars, 50 μm. **d** Comparisons of Angpt2 mRNA expression in fractionized adipocytes (Ad) and stromal vascular fraction (SVF) of SAT in WT mice. Each dot indicates a value obtained from one mouse and *n* = 4 mice/group pooled from two independent experiments. Horizontal bars indicate mean ± SD and *P* values versus SVF by two-tailed Student's t-test.

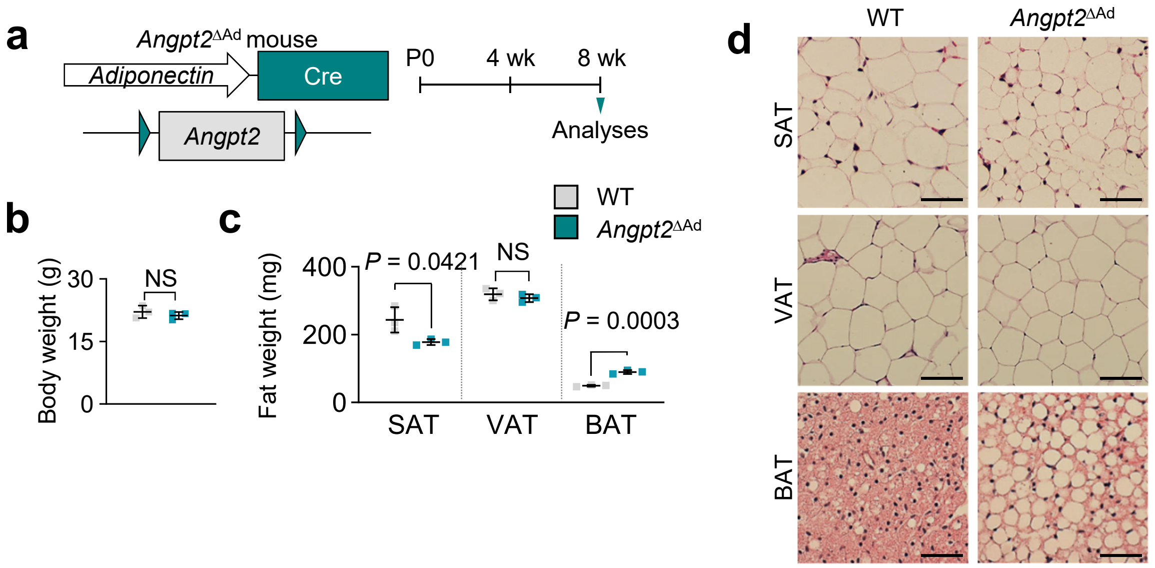

Supplemental Fig. 2 Congenital depletion of Angpt2 from adipocytes alters subcutaneous fat distribution
a Diagram for generation of *Angpt2*$^{\Delta Ad}$ mice, and their analyses at 8 weeks after birth. **b**, **c** Comparison of body weight and fat weight in different adipose tissues between WT and *Angpt2*$^{\Delta Ad}$ mice. Each dot indicates a value obtained from one mouse and $n = 3$ mice/group pooled from three independent experiments. Horizontal bars indicate mean ± SD and P values versus WT by two-tailed Student's t-test. NS, not significant. **d** Representative H&E-stained of different adipose tissues in WT and *Angpt2*$^{\Delta Ad}$ mice. Scale bars, 50 μm.

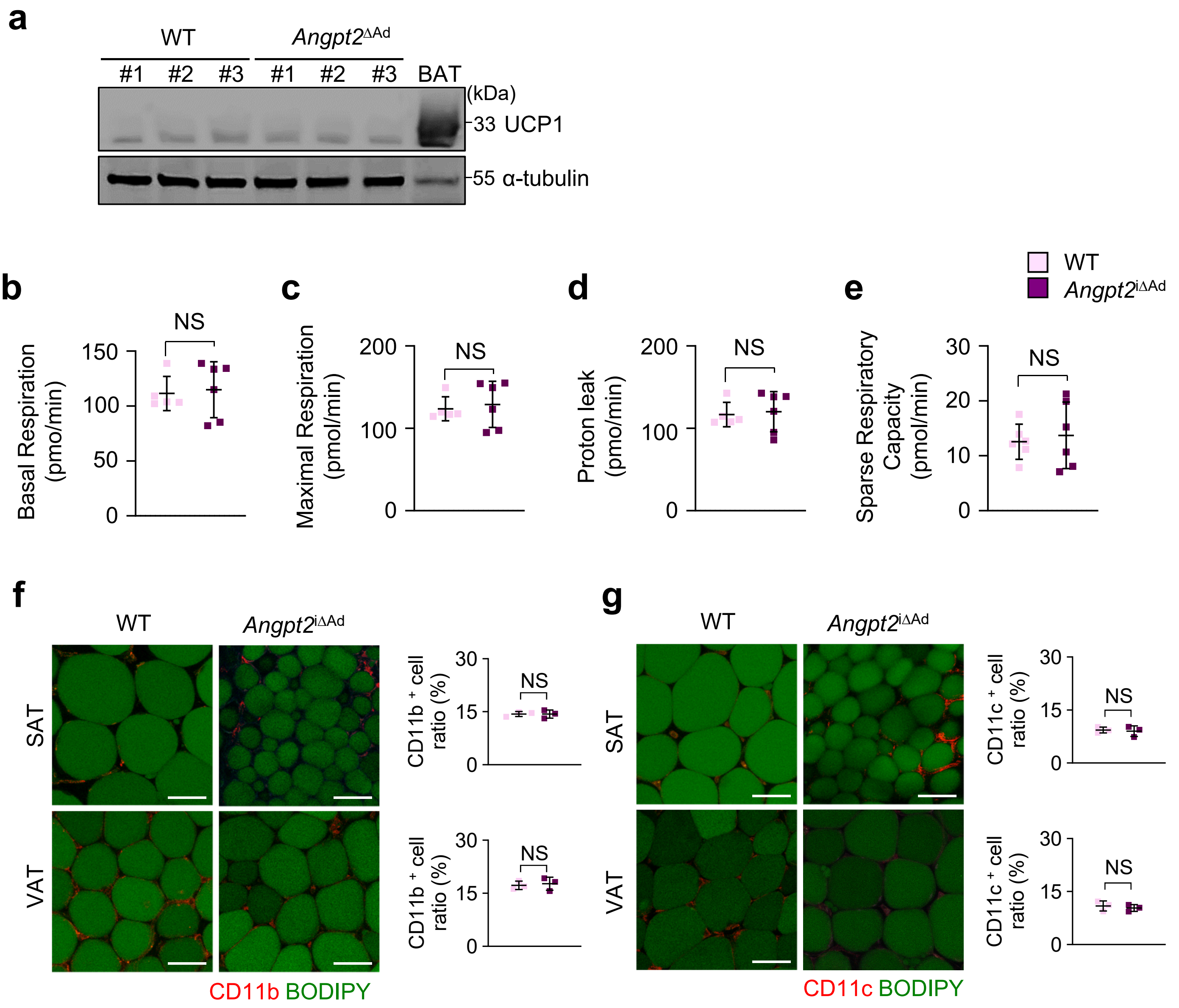

Supplemental Fig. 3 No apparent changes in oxygen consumption and immune cell infiltration in *Angpt2*$^{i\Delta Ad}$ mice

a Immunoblot analysis of indicated proteins in SAT of WT and *Angpt2*$^{i\Delta Ad}$ mice. BAT is used as a positive control. Samples derive from the same experiment and gels were processed in parallel. **b-e** Comparisons of basal respiration, maximal respiration, proton leak, and sparse respiratory capacity in primary cultured adipocytes isolated from SAT in WT and *Angpt2*$^{i\Delta Ad}$ mice. Each dot indicates a value obtained from one sample and n = 5 (WT), 6 (*Angpt2*$^{i\Delta Ad}$)/group. Horizontal bars indicate mean ± SD and P values versus WT by two-tailed Student's t-test. NS, not significant. **f**, **g** Representative images and comparison of indicated immune cell infiltration in SAT and VAT of WT and *Angpt2*$^{i\Delta Ad}$ mice. Magnified view is shown in right panels. Each dot indicates a value obtained from one mouse and *n* = 3 mice/group pooled from two independent experiments. Horizontal bars indicate mean ± SD and *P* values versus WT by two-tailed Student's t-test. NS, not significant. Scale bars, 30 μm.

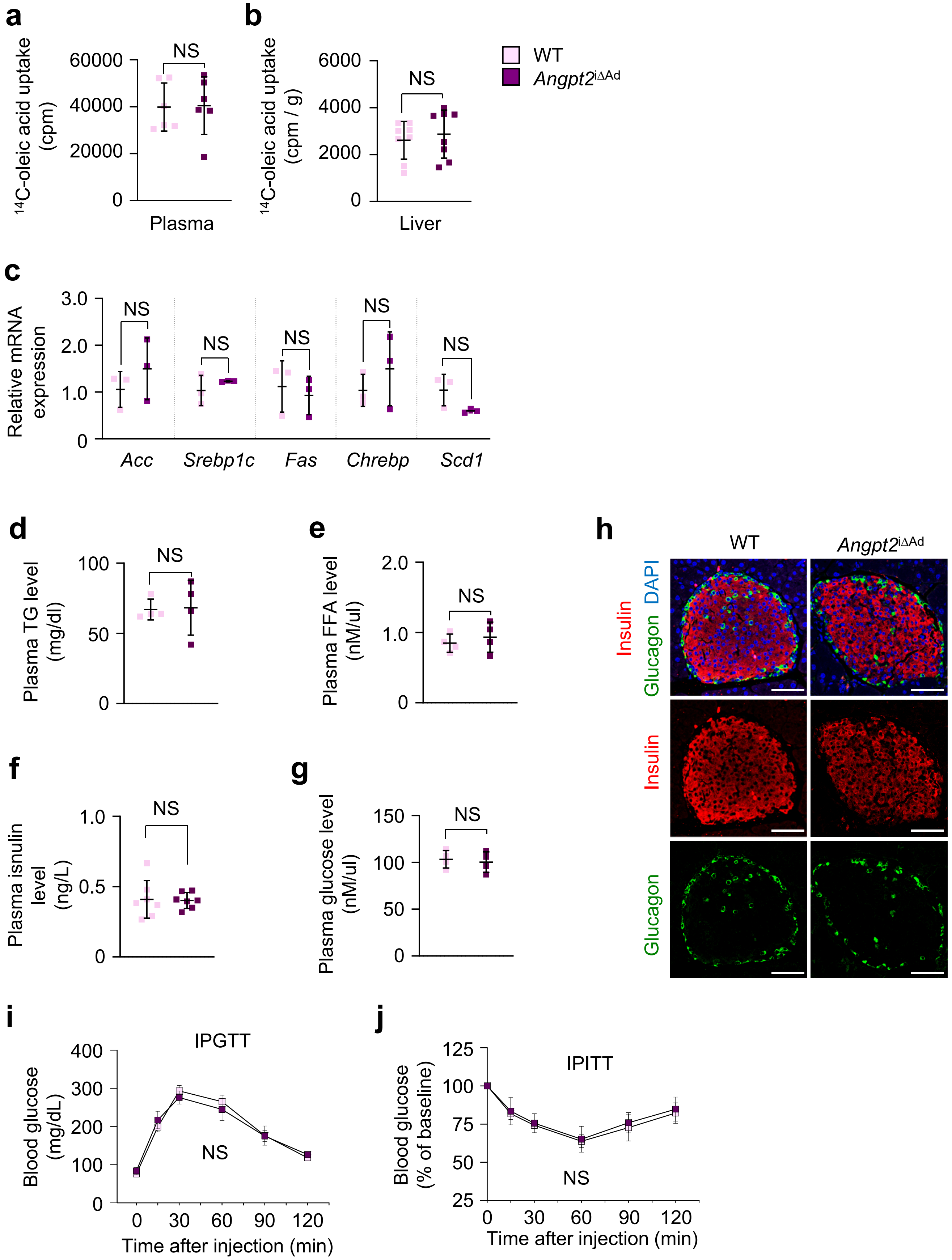
a
^{14}C-oleic acid uptake (cpm)
60000
40000
20000
0
NS
Plasma
b
^{14}C-oleic acid uptake (cpm / g)
6000
4000
2000
0
NS
Liver
WT
Angpt2$^{i\Delta Ad}$
c
Relative mRNA expression
3.0
2.0
1.0
0
NS
Acc
Srebp1c
Fas
Chrebp
Scd1
d
Plasma TG level (mg/dl)
100
50
0
NS
e
Plasma FFA level (nM/ul)
2.0
1.0
0
NS
h
WT
Angpt2$^{i\Delta Ad}$
Insulin Glucagon DAPI
Insulin
Glucagon
f
Plasma isnulin level (ng/L)
1.0
0.5
0
NS
g
Plasma glucose level (nM/ul)
150
100
50
0
NS
i
IPGTT
Blood glucose (mg/dL)
400
300
200
100
0
NS
0 30 60 90 120
Time after injection (min)
j
IPITT
Blood glucose (% of baseline)
125
100
75
50
25
NS
0 30 60 90 120
Time after injection (min)

Supplemental Fig. 4 No apparent changes in fatty acid production or uptake by liver, and systemic levels of insulin and glucose

a, **b** Comparisons of ^{14}C-oleic acid uptake in plasma and liver in WT and *Angpt2*$^{i\Delta Ad}$ mice. Each dot indicates a value obtained from one mouse using *n* =6 (plasma), 8 (liver) mice/group pooled from two independent experiments. Horizontal bars indicate mean ± SD and *P* values versus WT by two-tailed Student's t-test. NS, not significant. **c** Comparisons of indicated mRNA expression in SAT between WT and *Angpt2*$^{i\Delta Ad}$ mice under normal chow diet. Each dot indicates a value obtained from one mouse and n = 3 mice/group. Horizontal bars indicate mean ± SD and P values versus WT by two-tailed Student's t-test. NS, not significant. **d**, **e** Comparisons of plasma triglyceride (TG) and free fatty acid (FFA) levels between WT and *Angpt2*$^{i\Delta Ad}$ mice under normal chow diet. Each dot indicates a value obtained from one mouse and n = 4 mice/group pooled from two independent experiments. Horizontal bars indicate mean ± SD and P values versus WT by two-tailed Student's t-test. NS, not significant. **f**, **g** Comparison of plasma insulin and glucose levels between WT and *Angpt2*$^{i\Delta Ad}$ mice under normal chow diet. Each dot indicates a value obtained from one mouse and n = 7(insulin), n = 4(glucose) mice/group pooled from two independent experiments. Horizontal bars indicate mean ± SD and P values versus WT by two-tailed Student's t-test. NS, not significant. **h** Representative images of indicated proteins in pancreas of WT and *Angpt2*$^{i\Delta Ad}$ mice under normal chow diet. Scale bars, 100 µm. **i**, **j** Comparisons of intraperitoneal glucose tolerance test (IPGTT) and intraperitoneal insulin tolerance test (IPITT) between WT and *Angpt2*$^{i\Delta Ad}$ mice under normal chow diet. Each dot indicates a value obtained from one mouse and n = 6 mice/group pooled from three independent experiments. Horizontal bars indicate mean ± SEM and P values versus WT by two-tailed Student's t-test.

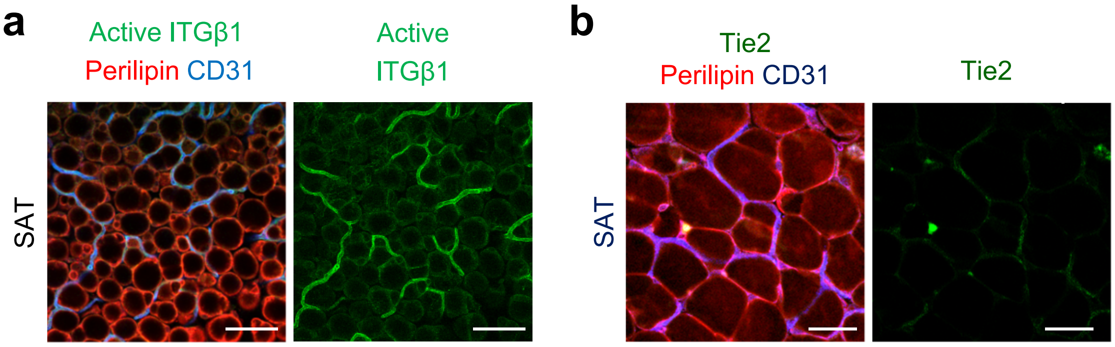

Supplemental Fig. 5 Expression of active ITGβ1 and Tie2 in ECs of SAT

a Representative images of active ITGβ1 expression in SAT of WT mice. Scale bars, 50 µm. **b** Representative images of Tie2 (antibody) expression in SAT of WT mice. Scale bars, 30 µm.

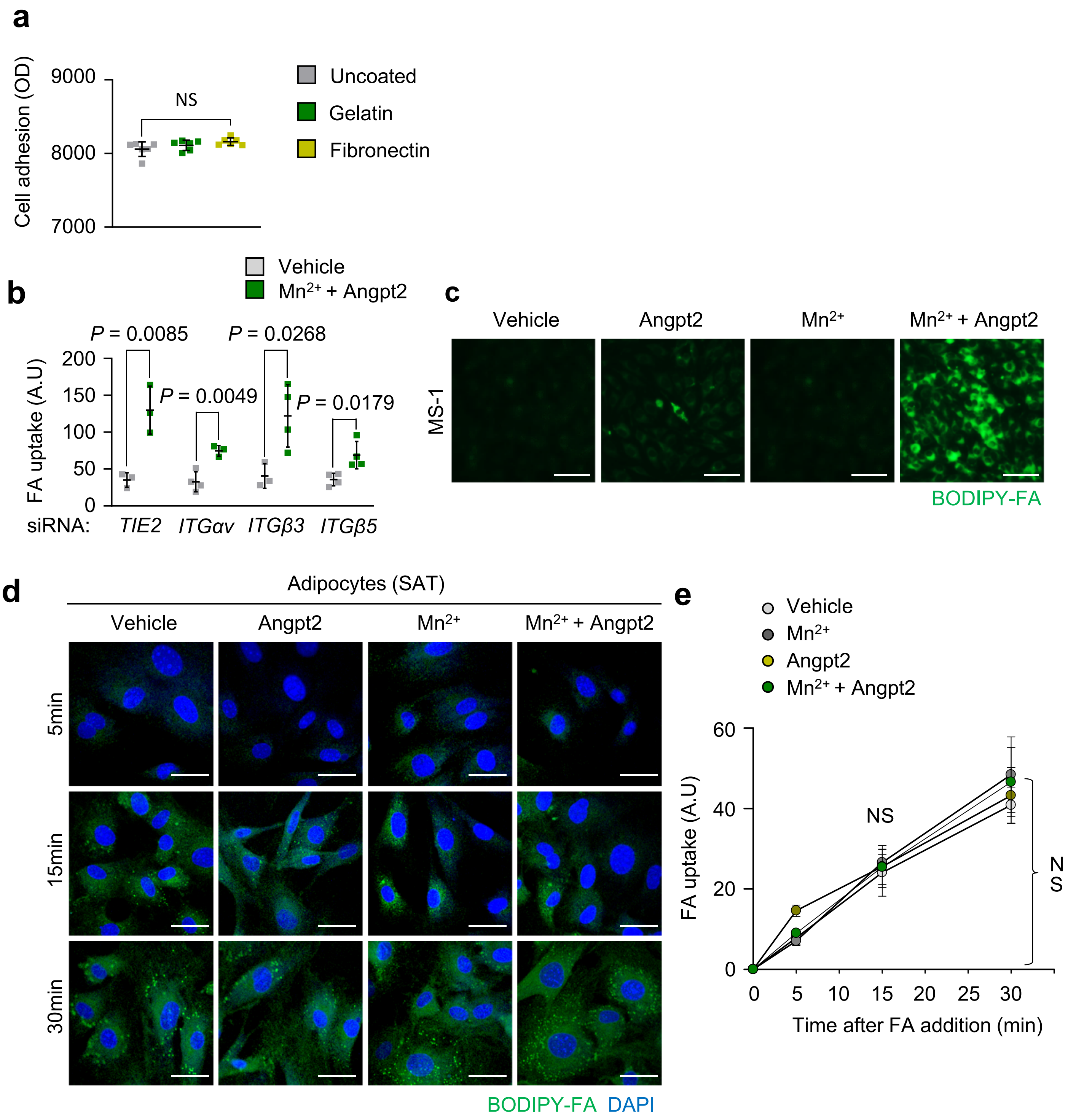

Supplemental Fig. 6 Angpt2 mediates endothelial FA uptake through ITGα5β1, not Tie2 or other integrins
a Comparison of cell adherence (OD) of HUVECs cultured on uncoated plates or those coated with 0.1% gelatin or 10 µg/mL fibronectin. Each dot indicates a value obtained from one well; *n* = 6/group pooled from two independent experiments. Horizontal bars indicate mean ± SD and *P* values versus Uncoated by two-tailed Student's t-test. NS, not significant. **b** Comparisons of FA (BODIPY C-12, 8uM) uptake after treatment with vehicle or Mn^{2+} (1mM) + Angpt2 (2.5ug/ml) for 15 min in siTie2 (n = 3), siITGαv (n = 4 and 3), siITGβ3 (n = 3 and 4) or siITGβ5 (n = 4) HUVECs. Horizontal bars indicate mean ± SD and *P* values versus vehicle by two-tailed Student's t-test. **c** Representative images of FA (BODIPY C-12) uptake after treatment with vehicle, Angpt2 (2.5ug/ml), Mn^{2+} (1mM), or Mn^{2+} (1mM) + Angpt2 (2.5ug/ml) for 15 min in mouse endothelial cells (MS-1). Scale bars, 100 µm. **d**, **e** Representative images and comparisons of FA (BODIPY C-12, 8uM) uptake after treatment with vehicle, Angpt2 (2.5ug/ml), Mn^{2+} (1mM), or Mn^{2+} (1mM) + Angpt2 (2.5ug/ml) for indicated time points in primary cultured adipocytes isolated from SAT of WT mice. Each group, n = 3. Horizontal bars indicate mean ± SD. NS, not significant. Scale bars, 30 µm.

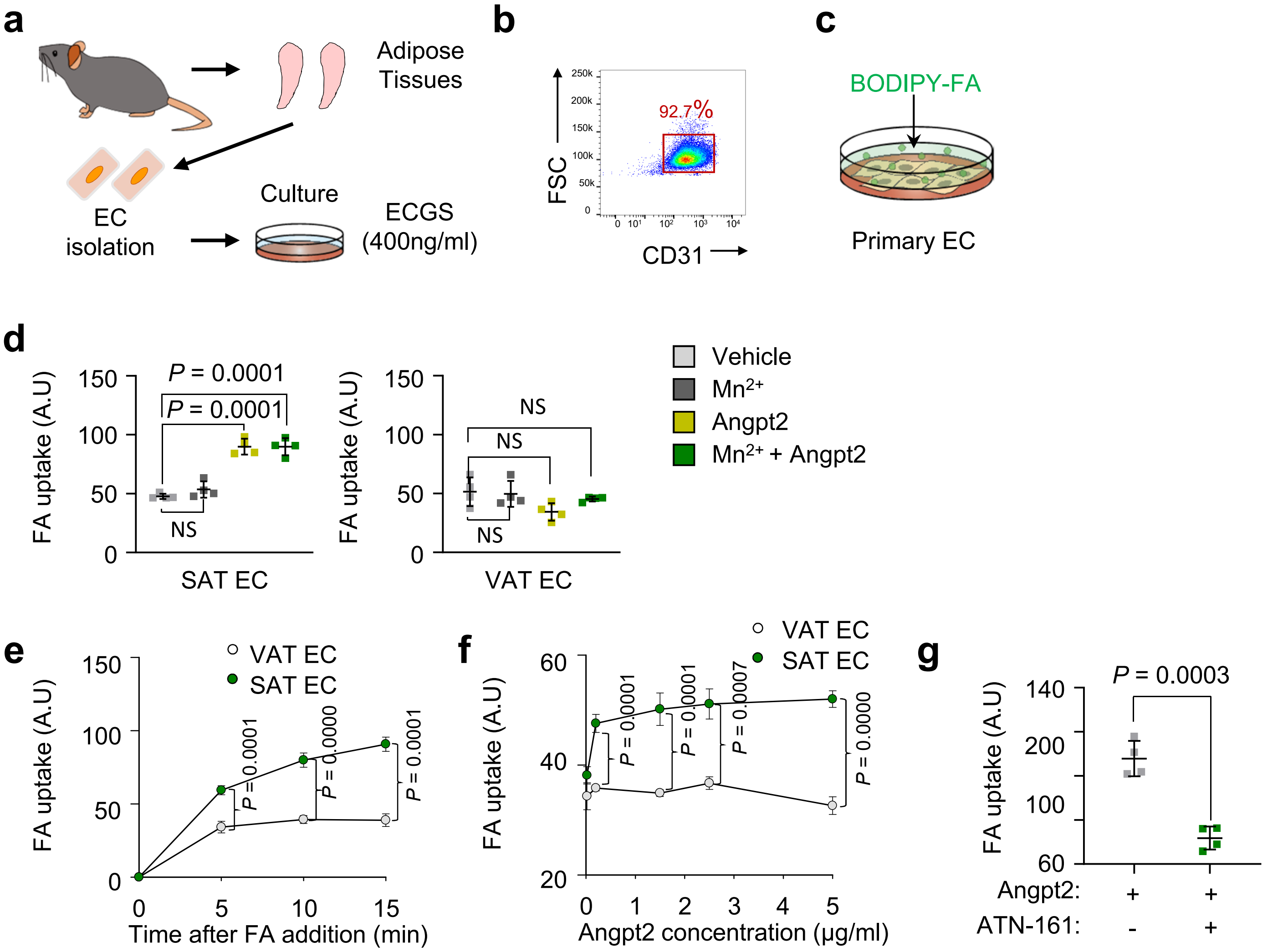

Supplemental Fig 7. Angpt2 stimulates FA uptake through integrin α5β1 signaling in primary cultured ECs from SAT

a-c Diagram depicting the FA uptake of primary cultured ECs from adipose tissues. Primary cultured ECs form WT mice were validated for it $CD31^{+}$ expression or Cre induction. **d** Comparison of FA (BODIPY C-12, 8uM) uptake after treatment with vehicle, Mn2+ (1mM), Angpt2 (2.5ug/ml) or Mn2+ (1mM) + Angpt2 (2.5ug/ml) for 15 min. **e** Comparisons of FA (BODIPY C-12, 8uM) uptake after treatment with Angpt2 (2.5ug/ml) for indicated time points and . n = 5 for each group. Horizontal bars indicate mean ± SEM and P values versus VAT EC by two-tailed Student's t-test. **f** Comparisons of FA (BODIPY C-12, 8uM) uptake after treatment with indicated concentration of Angpt2. n = 5 for each group. Horizontal bars indicate mean ± SEM and P values versus VAT EC by two-tailed Student's t-test. **g** Comparisons of FA (BODIPY C-12, 8uM) uptake after treatment with or without Angpt2 (2.5ug/ml), ITGα5β1 blocking peptide (ATN-161, 10uM) for 15min. Each dot indicates a mean of triplicate values from two independent experiments. Horizontal bars indicate mean ± SD and P values versus Angpt2 by two-tailed Student's t-test.

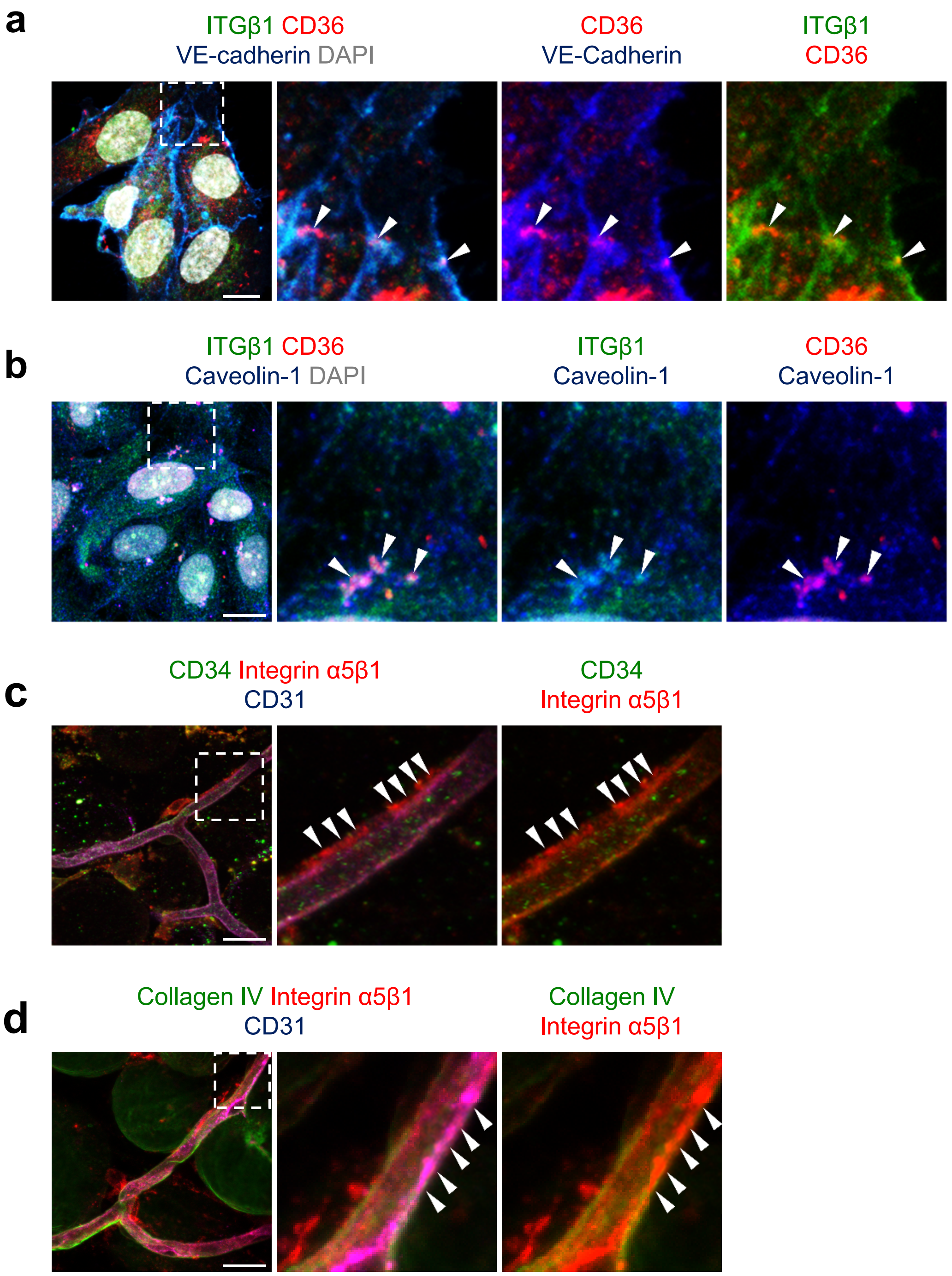

Supplemental Fig 8. Basolateral integrin α5β1 interacts with apical CD36 through transmembrane lipid rafts
a Representative images of ITGβ1, CD36, and VE-cadherin⁺ plasma membrane complex (arrowheads) in HUVECs treated with $Mn2^{+}$ (1mM) + Angpt2 (2.5ug/ml) for 15 min. Magnified view is shown in right panels. Scale bars, 10 μm. **b** Representative images of ITGβ1, CD36, and Caveolin-1 complex (arrowheads) in HUVECs treated with $Mn2^{+}$ (1mM) + Angpt2 (2.5ug/ml) for 15 min. Magnified view is shown in right panels. Scale bars, 10 μm. **c**, **d** Representative images of integrin α5β1 expression in collagen IV^{+} basolateral membrane (arrowheads), but not in $CD34^{+}$ apical membrane (arrowheads) of endothelial cells in SAT of WT mice. Magnified view is shown in right panels. Scale bars, 20 μm.

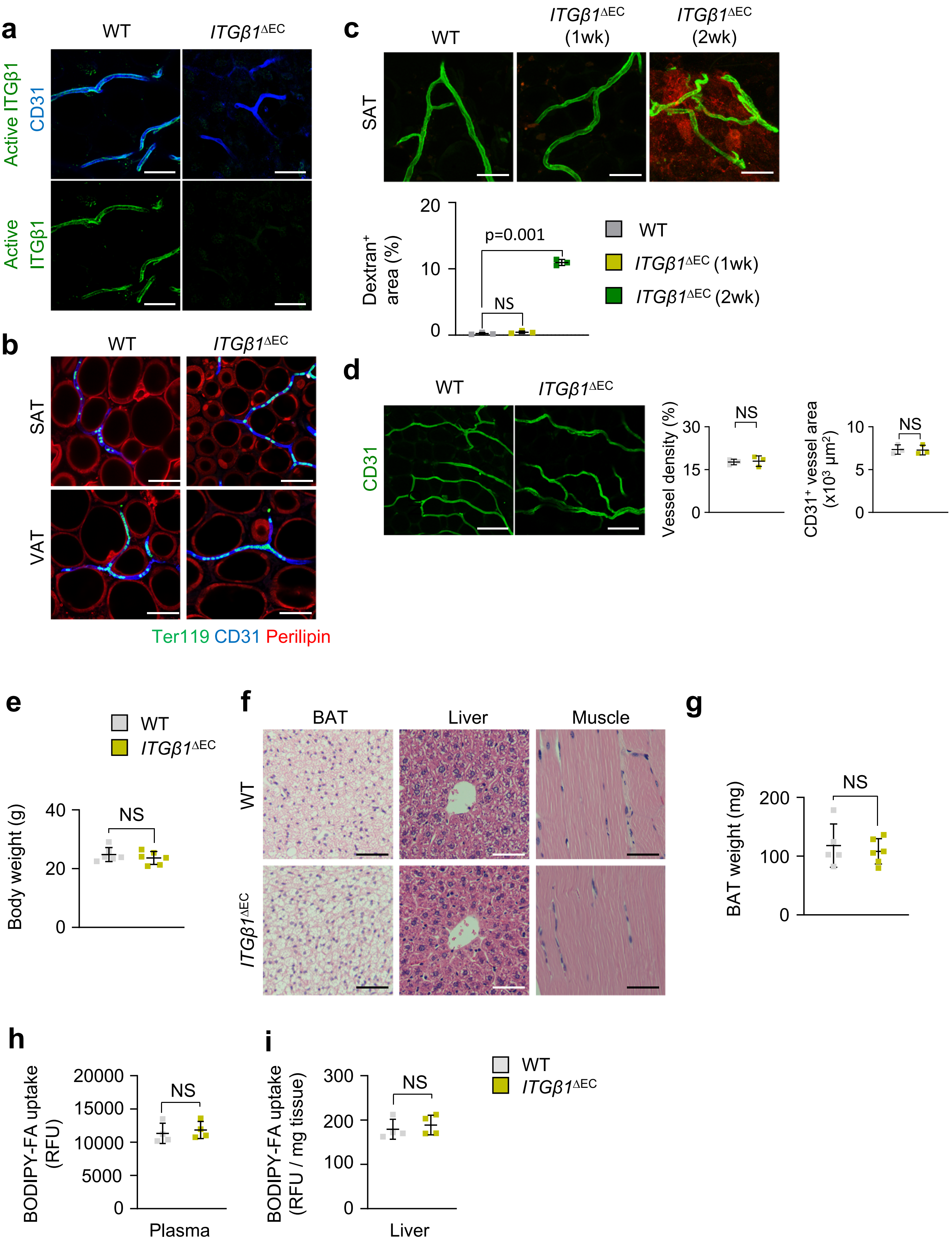

a
WT
ITGβ1ΔEC
Active ITGβ1 CD31
Active ITGβ1
b
WT
ITGβ1ΔEC
SAT
VAT
Ter119 CD31 Perilipin
c
WT
ITGβ1ΔEC (1wk)
ITGβ1ΔEC (2wk)
SAT
Dextran+ area (%)
p=0.001
NS
WT
ITGβ1ΔEC (1wk)
ITGβ1ΔEC (2wk)
d
WT
ITGβ1ΔEC
CD31
Vessel density (%)
NS
CD31+ vessel area (x10³ μm²)
NS
e
WT
ITGβ1ΔEC
Body weight (g)
NS
f
BAT
Liver
Muscle
WT
ITGβ1ΔEC
g
BAT weight (mg)
NS
h
BODIPY-FA uptake (RFU)
NS
Plasma
i
BODIPY-FA uptake (RFU / mg tissue)
NS
Liver
WT
ITGβ1ΔEC

Supplemental Fig 9. Preserved vascular integrity and density in *ITGβ1*$^{\Delta EC}$ mice through 1-week tamoxifen regimen

a Representative images of immunofluorescence staining of active ITGβ1 expression in EC of SAT in WT and *ITGβ1*$^{\Delta EC}$ mice. Scale bars, 50 μm. **b** Representative images of Ter119 expression in SAT and VAT in *ITGβ1*$^{\Delta EC}$ (1wk) mice. Scale bars, 50 μm. **c** Representative images (scale bars, 50 μm) and comparison of vascular permeability in ECs of SAT after FITC-dextran (kDa) injection in WT and *ITGβ1*$^{\Delta EC}$ mice with different tamoxifen regimen. Each dot indicates a value obtained from one mouse and $n = 3$ mice/group pooled from two independent experiments. **d** Representative images and comparison of vascular density and $CD31^+$ vessel area in SAT of WT and *ITGβ1*$^{\Delta EC}$ mice. Each dot indicates a value obtained from one mouse; $n = 3$ mice/group pooled from two independent experiments. Horizontal bars indicate mean ± SD and *P* values versus WT by two-tailed Student's t-test. NS, not significant. Scale bars, 70 μm. **e** Comparison of body weight between WT and *ITGβ1*$^{\Delta EC}$ mice. Each dot indicates a value obtained from one mouse and $n = 6$ mice/group pooled from three independent experiments. Horizontal bars indicate mean ± SD. NS, not significant. **f** Representative H&E-stained images of indicated organs of WT and *ITGβ1*$^{\Delta EC}$ mice. Scale bars, 50 μm. **g** Comparisons of weight of BAT between WT and *ITG*β*1*$^{\Delta EC}$ mice. Each dot indicates a value obtained from one mouse and $n = 6$ mice/group pooled from three independent experiments. Horizontal bars indicate mean ± SD. NS, not significant. Representative images of immunofluorescence staining of active ITGβ1 expression in EC of SAT in WT and *ITGβ1*$^{\Delta EC}$ mice. Scale bars, 50 μm. **h**, **i** Comparisons of FA (BODIPY C-16, 0.5g/kg) uptake into plasma and liver between WT and *ITGβ1*$^{\Delta EC}$ mice. Each dot indicates a mean of quadruplicate values from three independent experiments. Horizontal bars indicate mean ± SD and *P* value versus vehicle by two-tailed Student's t-test. NS, not significant.

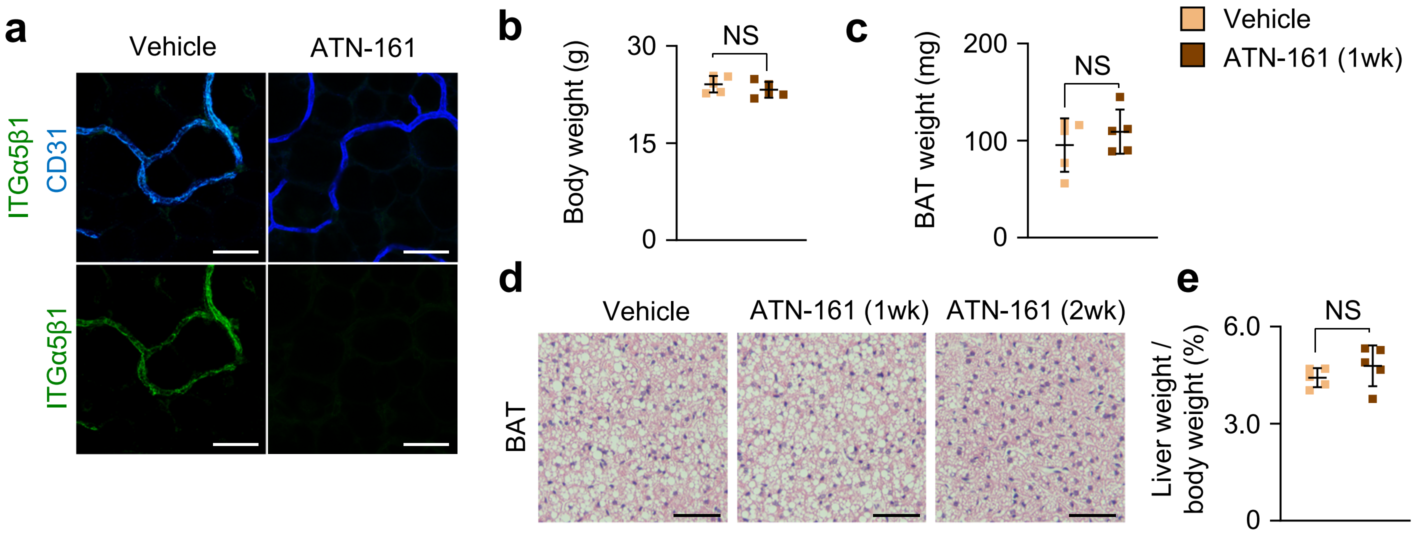

Supplemental Fig 10. Integrin α5β1 does not affect FA transport in other organs
a Representative images of immunofluorescence staining of integrin α5β1 expression in EC of SAT in mice treated with vehicle or ATN-161 (30mg/kg) for 1wk. Scale bars, 50 μm. **b** Comparison of body weight between vehicle and integrin α5β1 blocking peptide (ATN-161, 30mg/kg) treated mice. Each dot indicates a value obtained from one mouse and $n = 5$ mice/group pooled from three independent experiments. Horizontal bars indicate mean ± SD. NS, not significant. **c** Comparison of BAT weight between vehicle and ATN-161 treated mice. Each dot indicates a value obtained from one mouse and $n = 5$ mice/group pooled from three independent experiments. Horizontal bars indicate mean ± SD. NS, not significant. **d** H&E-stained images BAT between vehicle and ATN-161 treated mice. Scale bars, 50 μm. **e** Comparison of liver-to-body weight ratio between vehicle and ATN-161 treated mice. Each dot indicates a value obtained from one mouse and $n = 5$ mice/group pooled from three independent experiments. Horizontal bars indicate mean ± SD. NS, not significant.

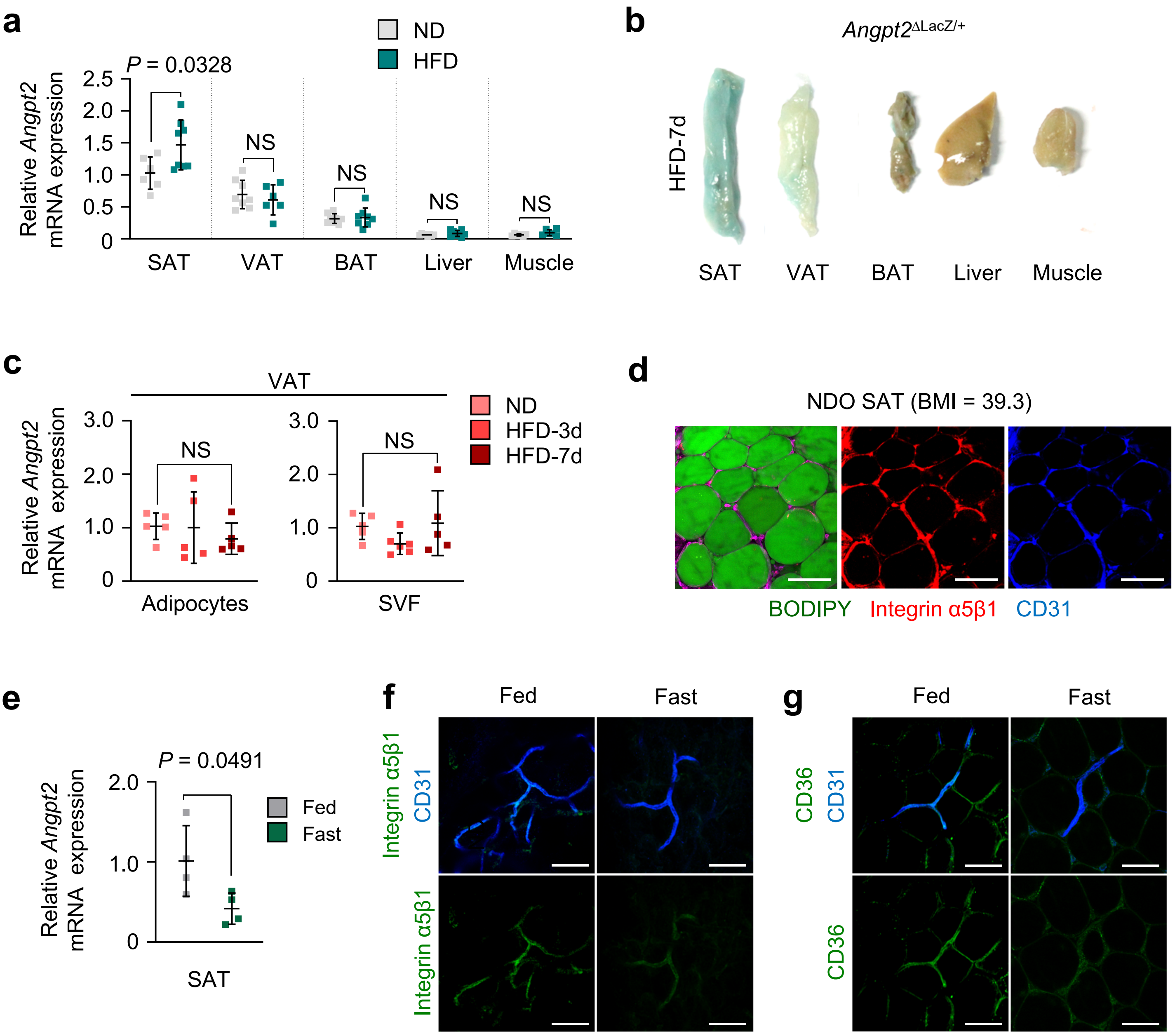

Supplemental Fig. 11 Angpt2- integrin α5β1 expression is correlated with metabolic adaptation
a Comparisons of Angpt2 mRNA expression in in different adipose tissues and metabolic organs of WT mice fed with HFD for 3 months. Each dot indicates a value obtained from one mouse and *n* = 6 except 8 (VAT ND, BAT, and Liver; SAT, VAT, BAT, and Liver HFD) mice/group pooled from three independent experiments. Horizontal bars indicate mean ± SD and *P* values versus ND by two-tailed Student's t-test. NS, not significant. **b** Representative images of β-galactosidase activity reflecting Angpt2 expression in different adipose tissues and metabolic organs of *Angpt2*$^{\Delta LacZ/+}$ reporter mice fed with HFD for 7 days. **c** Comparisons of Angpt2 mRNA expression in fractionated adipocytes and SVF in VAT of WT mice fed with HFD for 3 days and 7 days. Each dot indicates a value obtained from one mouse and *n* = 5 except 6 (SVF HFD-7d) mice/group pooled from two independent experiments. Horizontal bars indicate mean ± SD and *P* values versus ND by one-way ANOVA followed by Tukey's multiple comparison test. NS, not significant. **d** Representative images of immunofluorescence staining of integrin α5β1 expression in EC of SAT in non-diabetic obese human (body mass index :39.3). Scale bars, 50 μm. **e** Comparisons of Angpt2 mRNA expression in SAT during fed/fast cycle in WT mice. Each dot indicates a value obtained from one mouse and n = 4 mice/group. Horizontal bars indicate mean ± SD and P values versus WT by two-tailed Student's t-test. **f** Representative images of integrin α5β1 expression in SAT during fed/fast cycle in WT mice. Scale bars, 50 μm. **g** Representative images of CD36 expression in SAT during fed/fast cycle in WT mice. Scale bars, 50 μm.

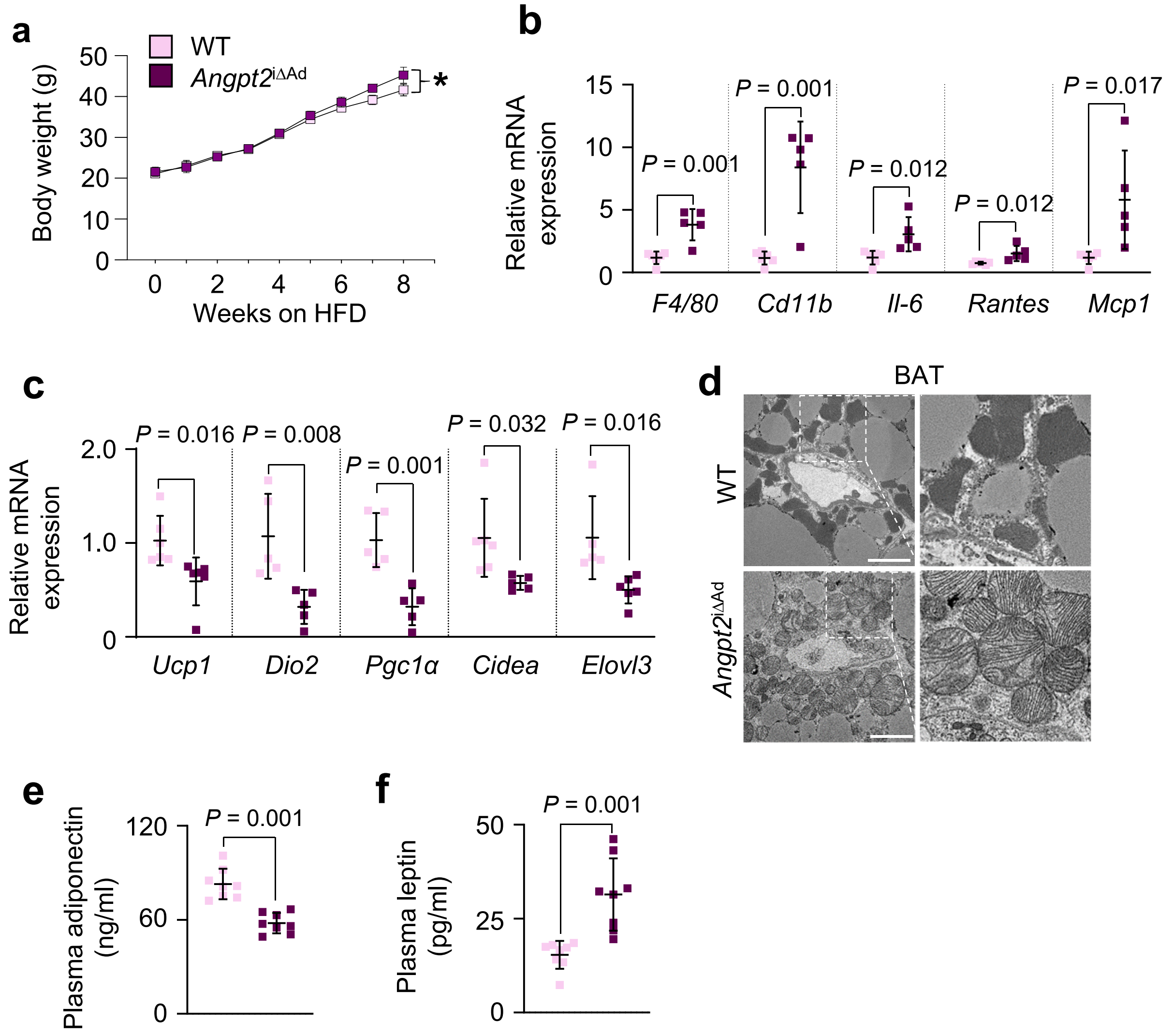

Supplemental Fig. 12 Adipocyte-derived Angpt2 prevents metabolic complications associated with obesity
a Comparison of body weight changes between WT and *Angpt2*$^{i\Delta Ad}$ mice fed with HFD. Each dot indicates a value obtained from one mouse; n = 6 (*Angpt2*$^{i\Delta Ad}$) or 8 (WT) mice/group pooled from three independent experiments. Horizontal bars indicate mean ± SD and **P* < 0.05 versus WT by two-tailed Student's t-test. *P* = 0.0175 (6wk), 0.0011 (7wl). 0.0105 (8wk). **b** Comparisons of indicated mRNA expression in VAT between WT and *Angpt2*$^{i\Delta Ad}$ mice. Each dot indicates a value obtained from one mouse; n = 5 (*Angpt2*$^{i\Delta Ad}$) or 6 (WT) mice/group. Horizontal bars indicate mean ± SD and P values versus WT by two-tailed Student's t-test. **c** Comparisons of indicated mRNA expression in BAT between WT and *Angpt2*$^{i\Delta Ad}$ mice. Each dot indicates a value obtained from one mouse; n = 5 except 6 (all Ucp1, Cidea WT, and Elovl3 *Angpt2*$^{i\Delta Ad}$) mice/group. Horizontal bars indicate mean ± SD and P values versus WT by two-tailed Student's t-test. **d** Representative electron microspore images indicating mitochondrial morphology in BAT of WT and *Angpt2*$^{i\Delta Ad}$ mice. Magnified view is shown in right panels. Scale bars, 2 µm. **e**, **f** Comparison of plasma adiponectin and leptin levels between WT and *Angpt2*$^{i\Delta Ad}$ mice. Each dot indicates a value obtained from one mouse; n = 8 mice/group pooled from two independent experiments. Horizontal bars indicate mean ± SD and *P* values versus WT by two-tailed Student's t-test. NS, not significant

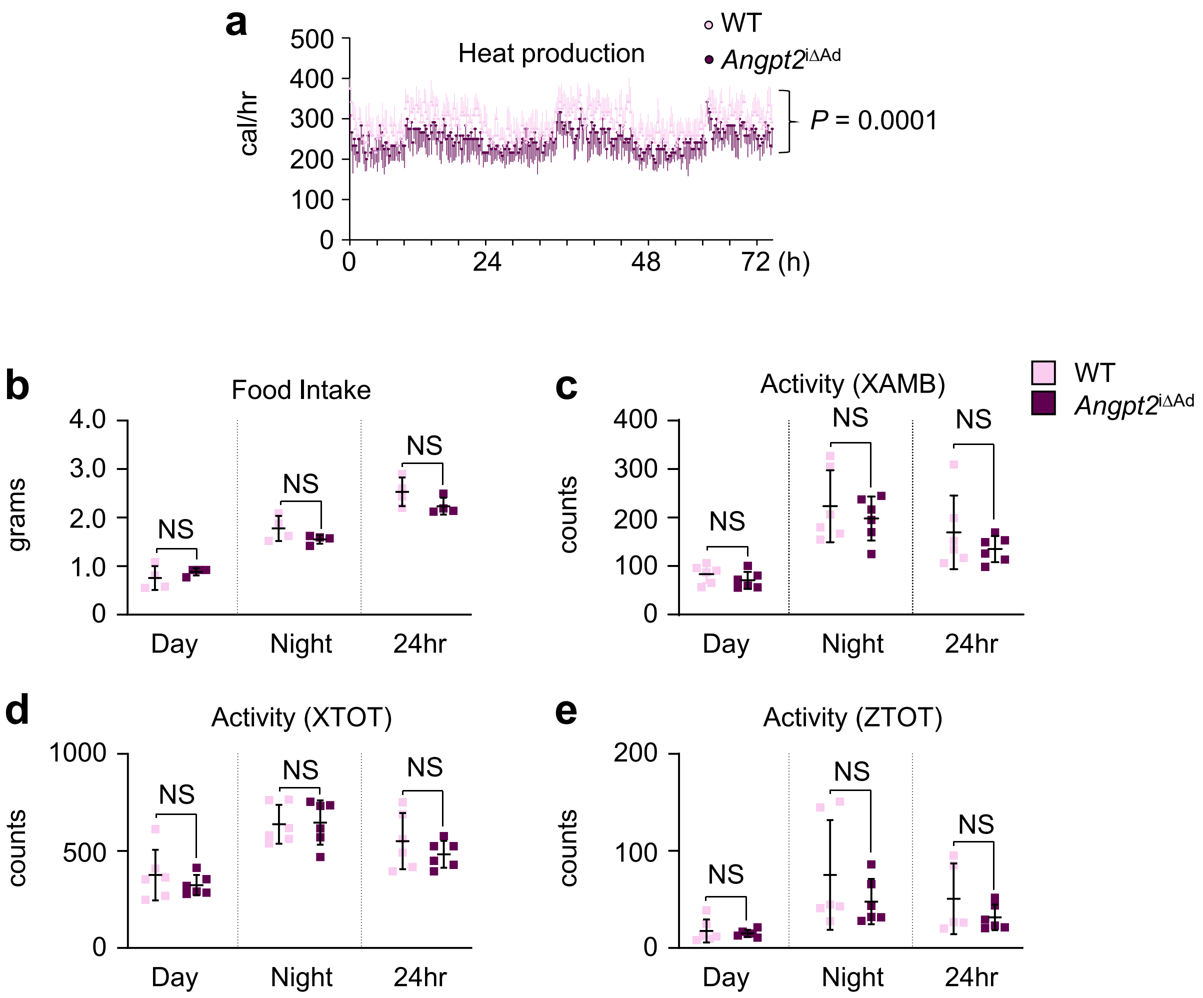

Supplemental Fig. 13 Adipocyte-derived Angpt2 affects metabolic activity during high fat diet treatment

a-e 8-week-old mice of WT and *Angpt2*$^{i\Delta Ad}$ were subjected to metabolic cage assessment with CLAMS analysis. Comparisons of heat production, food intake, and activity (XAMB = ambulatory activity on the X axis, XTOT = total beam breaks on the X axis, ZTOT = total beam breaks on the Z axis). Each dot indicates a value obtained from one mouse and $n = 6$ except 4 (food intake) mice/group pooled from three independent experiments. Horizontal bars indicate mean ± SD and *P* values versus WT by two-tailed Student's t-test. NS, not significant.

Supplementary Table 1. List of Gene Signatures Enriched in SAT of NDO

Symbol	Entrez Gene Name	Type
ACSM5	acyl-CoA synthetase medium-chain family member 5	enzyme
ANGPT2	angiopoietin 2	secretory molecule
ATOH8	atonal bHLH transcription factor 8	transcription regulator
BRWD1	bromodomain and WD repeat domain containing 1	transcription regulator
C8orf59	chromosome 8 open reading frame 59	other
CDO1	cysteine dioxygenase type 1	enzyme
CPM	carboxypeptidase M	peptidase
CTC-338M12.4	uncharacterized LOC101928649	other
FAM13A	family with sequence similarity 13 member A	other
FAM199X	family with sequence similarity 199, X-linked	other
FIBIN	fin bud initiation factor homolog (zebrafish)	other
GBP4	guanylate binding protein 4	enzyme
GPM6B	glycoprotein M6B	other
HADH	hydroxyacyl-CoA dehydrogenase	enzyme
ID4	inhibitor of DNA binding 4, HLH protein	transcription regulator
ING3	inhibitor of growth family member 3	other
ITGBL1	integrin subunit beta like 1	other
LARP1B	La ribonucleoprotein domain family member 1B	other
LOC283788	FSHD region gene 1 pseudogene	other
MAZ	MYC associated zinc finger protein	transcription regulator
MRPL2	mitochondrial ribosomal protein L2	other
NCKAP1	NCK associated protein 1	other
PML	promyelocytic leukemia	transcription regulator
RC3H2	ring finger and CCCH-type domains 2	enzyme
RIOK3	RIO kinase 3	kinase
RNF180	ring finger protein 180	enzyme
SDHC	succinate dehydrogenase complex subunit C	enzyme
SHISA3	shisa family member 3	other
TACC2	transforming acidic coiled-coil containing protein 2	other
TBX5-AS1	TBX5 antisense RNA 1	other
THAP6	THAP domain containing 6	other
YPEL2	yippee like 2	other
ZFYVE16	zinc finger FYVE-type containing 16	transporter
ZNF41	zinc finger protein 41	other

Supplementary Table 2. Comparison Analysis of Expression Value of Angpt2 Between NDO vs DO

Reference	Sample	Organ	Angpt2 (log2)	Diabetic	Obese
GSE20950	GSM523846	SAT	5.68443	**No**	**Yes**
	GSM523847	SAT	5.96895	**No**	**Yes**
	GSM523848	SAT	5.41435	**No**	**Yes**
	GSM523850	SAT	6.02601	**No**	**Yes**
	GSM523851	SAT	6.20049	**No**	**Yes**
	GSM523852	SAT	5.29078	**No**	**Yes**
	GSM523854	SAT	5.42732	**No**	**Yes**
	GSM523855	SAT	5.29331	**No**	**Yes**
	GSM523849	SAT	6.24495	**No**	**Yes**
	GSM523853	SAT	5.23687	**No**	**Yes**
	GSM523838	SAT	5.41725	**Yes**	**Yes**
	GSM523842	SAT	4.65797	**Yes**	**Yes**
	GSM523843	SAT	3.81677	**Yes**	**Yes**
	GSM523844	SAT	4.16805	**Yes**	**Yes**
	GSM523837	SAT	5.74503	**Yes**	**Yes**
	GSM523839	SAT	4.70385	**Yes**	**Yes**
	GSM523840	SAT	5.10448	**Yes**	**Yes**
	GSM523841	SAT	4.76821	**Yes**	**Yes**
	GSM523845	SAT	4.40654	**Yes**	**Yes**
GSE20950	GSM523866	VAT	5.21653	**No**	**Yes**
	GSM523867	VAT	5.47016	**No**	**Yes**
	GSM523868	VAT	5.54625	**No**	**Yes**
	GSM523870	VAT	5.91081	**No**	**Yes**
	GSM523871	VAT	5.39194	**No**	**Yes**
	GSM523872	VAT	5.44931	**No**	**Yes**
	GSM523874	VAT	5.42942	**No**	**Yes**
	GSM523875	VAT	4.96206	**No**	**Yes**
	GSM523869	VAT	5.14822	**No**	**Yes**
	GSM523873	VAT	5.00898	**No**	**Yes**
	GSM523856	VAT	5.8045	**Yes**	**Yes**
	GSM523857	VAT	5.96266	**Yes**	**Yes**
	GSM523859	VAT	4.99983	**Yes**	**Yes**
	GSM523860	VAT	5.37129	**Yes**	**Yes**
	GSM523861	VAT	5.3398	**Yes**	**Yes**
	GSM523865	VAT	4.91112	**Yes**	**Yes**
	GSM523858	VAT	5.76148	**Yes**	**Yes**
	GSM523862	VAT	5.32854	**Yes**	**Yes**
	GSM523863	VAT	4.14541	**Yes**	**Yes**
	GSM523864	VAT	4.71308	**Yes**	**Yes**

Supplementary Table 2. Comparison Analysis of Expression Value of Angpt2 Between NDO vs DO (continued)

Reference	Sample	Organ	Angpt2 (log2)	Diabetic	Obese
GSE29226	GSM722747	SAT	5.842176832	**Yes**	**Yes**
	GSM722748	SAT	5.96256059	**Yes**	**Yes**
	GSM722749	SAT	7.009671885	**Yes**	**Yes**
	GSM722750	SAT	7.24862022	**Yes**	**Yes**
	GSM722751	SAT	6.950176766	**Yes**	**Yes**
	GSM722752	SAT	6.815601045	**Yes**	**Yes**
	GSM722753	SAT	5.84192533	**Yes**	**Yes**
	GSM722754	SAT	5.829996663	**Yes**	**Yes**
	GSM722755	SAT	7.138712094	**Yes**	**Yes**
	GSM722756	SAT	7.094584454	**Yes**	**Yes**
	GSM722757	SAT	7.165801422	**Yes**	**Yes**
	GSM722758	SAT	6.882251757	**Yes**	**Yes**
	GSM722735	SAT	6.887110924	**No**	**Yes**
	GSM722736	SAT	6.898571158	**No**	**Yes**
	GSM722737	SAT	7.185608898	**No**	**Yes**
	GSM722738	SAT	7.233466292	**No**	**Yes**
	GSM722739	SAT	7.430469278	**No**	**Yes**
	GSM722740	SAT	7.281058353	**No**	**Yes**
	GSM722741	SAT	7.207180306	**No**	**Yes**
	GSM722742	SAT	6.949231422	**No**	**Yes**
	GSM722743	SAT	7.421837618	**No**	**Yes**
	GSM722744	SAT	7.519109396	**No**	**Yes**
	GSM722745	SAT	7.4956472	**No**	**Yes**
	GSM722746	SAT	7.503929068	**No**	**Yes**
GSE29231	GSM722960	VAT	8.749886183	**Yes**	**Yes**
	GSM722961	VAT	8.740134912	**Yes**	**Yes**
	GSM722962	VAT	7.494639614	**Yes**	**Yes**
	GSM722963	VAT	7.497875782	**Yes**	**Yes**
	GSM722964	VAT	6.377987858	**Yes**	**Yes**
	GSM722965	VAT	6.327888523	**Yes**	**Yes**
	GSM722966	VAT	8.526636166	**Yes**	**Yes**
	GSM722967	VAT	8.729505187	**Yes**	**Yes**
	GSM722968	VAT	7.558612039	**Yes**	**Yes**
	GSM722969	VAT	7.617063344	**Yes**	**Yes**
	GSM722970	VAT	6.146129755	**Yes**	**Yes**
	GSM722971	VAT	6.054770351	**Yes**	**Yes**
	GSM722948	VAT	7.199662531	**No**	**Yes**
	GSM722949	VAT	7.420415029	**No**	**Yes**
	GSM722950	VAT	7.004905829	**No**	**Yes**
	GSM722951	VAT	6.821506142	**No**	**Yes**
	GSM722952	VAT	6.55152217	**No**	**Yes**
	GSM722953	VAT	6.892002369	**No**	**Yes**
	GSM722954	VAT	6.926888964	**No**	**Yes**
	GSM722955	VAT	7.123738611	**No**	**Yes**
	GSM722956	VAT	7.068681558	**No**	**Yes**
	GSM722957	VAT	6.83607561	**No**	**Yes**
	GSM722958	VAT	6.49567278	**No**	**Yes**
	GSM722959	VAT	6.416518882	**No**	**Yes**

Supplementary Table 2. Comparison Analysis of Expression Value of Angpt2 Between NDO vs DO (continued)

Reference	Sample	Organ	Angpt2 (log2)	Diabetic	Obese
GSE16415	GSM412612	VAT	0.803238	**Yes**	**Yes**
	GSM412613	VAT	2.35229	**Yes**	**Yes**
	GSM412614	VAT	3.20733	**Yes**	**Yes**
	GSM412615	VAT	1.87805	**Yes**	**Yes**
	GSM412616	VAT	0.865801	**Yes**	**Yes**
	GSM412607	VAT	0.68297	**No**	**Yes**
	GSM412608	VAT	1.1199	**No**	**Yes**
	GSM412609	VAT	1.36652	**No**	**Yes**
	GSM412610	VAT	0.933313	**No**	**Yes**
	GSM412611	VAT	0.636108	**No**	**Yes**
GSE71416	GSM1833926	VAT	4.93041	**Yes**	**Yes**
	GSM1833927	VAT	4.20072	**Yes**	**Yes**
	GSM1833928	VAT	6.18198	**Yes**	**Yes**
	GSM1833929	VAT	5.23973	**Yes**	**Yes**
	GSM1833930	VAT	5.25077	**Yes**	**Yes**
	GSM1833931	VAT	6.0778	**Yes**	**Yes**
	GSM1833932	VAT	5.93928	**Yes**	**Yes**
	GSM1833933	VAT	6.32325	**Yes**	**Yes**
	GSM1833934	VAT	6.21249	**Yes**	**Yes**
	GSM1833935	VAT	5.97403	**Yes**	**Yes**
	GSM1833936	VAT	6.08283	**Yes**	**Yes**
	GSM1833937	VAT	3.26154	**Yes**	**Yes**
	GSM1833938	VAT	4.64061	**Yes**	**Yes**
	GSM1833939	VAT	6.46176	**Yes**	**Yes**
	GSM1833940	VAT	5.91735	**No**	**Yes**
	GSM1833941	VAT	4.42704	**No**	**Yes**
	GSM1833942	VAT	6.40622	**No**	**Yes**
	GSM1833943	VAT	4.87726	**No**	**Yes**
	GSM1833944	VAT	5.70147	**No**	**Yes**
	GSM1833945	VAT	6.18053	**No**	**Yes**

Supplementary Table 3. Primer Sets for Quantitative RT-PCR

Name	Sequence (5'-3')	
Mouse *Gapdh*	Forward	AATGTGTCCGTCGTGGATCT
	Reverse	CATCGAAGGTGGAAGAGTGG
Mouse *Angpt2*	Forward	CTCCAAGAGCTCGGTTGCTATCCG
	Reverse	GGCCTTGATCTCCTCTGTGGAGTTG
Mouse *Nfatc1*	Forward	CCTCTGTGAGTCTTTGGGTTAG
	Reverse	ACCACGGCAGGCTTATTT
Mouse *Foxc2*	Forward	TCTGTAAACGAGTGCGGATTT
	Reverse	TGGGCAAGACGAAACCTTATC
Mouse *Perilipin*	Forward	GTGCAATGCCTATGAGAAGGGTGTAC
	Reverse	GTAGAGATGGTGCCCTTCAGTTCAGA
Mouse *Pecam*	Forward	CAGTCCGAAAATGGAACTGCAC
	Reverse	TGAGCCCTACCTTTGCTTCAC
Mouse *Ucp1*	Forward	ACTGCCACACCTCCAGTCATT
	Reverse	CTTTGCCTCACTCAGGATTGG
Mouse *Dio2*	Forward	CAGTGTGGTGCACGTCTCCAATC
	Reverse	TGAACCAAAGTTGACCACCAG
Mouse *Pgc1α*	Forward	TGATGTGAATGACTTGGATACAGACA
	Reverse	GCTCATTGTTGTACTGGTTGGATATG
Mouse *Cidea*	Forward	TGCTCTTCTGTATCGCCCAGT
	Reverse	GCCGTGTTAAGGAATCTGCTG
Mouse *Elovl3*	Forward	TCCGCGTTCTCATGTAGGTCT
	Reverse	GGACCTGATGCAACCCTATGA
Mouse *Cd11b*	Forward	TGGCCTATACAAGCTTGGCTTT
	Reverse	AAAGGCCGTTACTGAGGTGG
Mouse *F480*	Forward	CTTTGGCTATGGGCTTCCAGTC
	Reverse	GCAAGGAGGACAGAGTTTATCGTG
Mouse *Il-6*	Forward	GCTACCAAACTGGATATAATCAGGA
	Reverse	CCAGGTAGCTATGGTACTCCAGAA
Mouse *Rantes*	Forward	GCAAGTGCTCCAATCTTGCA
	Reverse	CTTGGCGGTTCCTTCGAGT
Mouse *Mcp1*	Forward	AGGTCCCTGTCATGCTTGTG
	Reverse	TCTGGACCCATTCCTTCTTG

Supplementary Table 3. Primer Sets for Quantitative RT-PCR (continued)

Name	Sequence (5'-3')	
Mouse *36b4*	Forward	gaggaatcagatgaggatatggga
	Reverse	aagcaggctgacttggttgc
Mouse *Acc1*	Forward	ggacagactgatcgcagagaaag
	Reverse	tggagagccccacacaca
Mouse *Chrebp*	Forward	gaaacctgaggctgtcatcct
	Reverse	cgtggtattcgcgcatca
Mouse *Fas*	Forward	gctgcggaaacttcaggaaat
	Reverse	agagacgtgtcactcctggactt
Mouse Scd1	Forward	ccggagacccccttagatcga
	Reverse	tagcctgtaaaagatttctgcaaacc
Mouse *Srebp-1c*	Forward	ggagccatggattgcacatt
	Reverse	ggcccgggaagtcactgt
Human *GAPDH*	Forward	CCACTCCTCCACCTTTGACG
	Reverse	TTCGTTGTCATACCAGGAAATGAG
Human *FATP3*	Forward	AGAGACCTTCAAACAGCAGAAAG
	Reverse	GTCCAGAACGTACAGTGGGT
Human *FATP4*	Forward	AGGCAAAGGTGCGACAGTG
	Reverse	CCAGTGGGTATCTGTGCCC
Human *CD36*	Forward	GCCAAGGAAAATGTAACCCAGG
	Reverse	GCCTCTGTTCCAACTGATAGTGA
Human *ANGPT2*	Forward	TGCCACGGTGAATAATTCAG
	Reverse	TTCTTCTTTAGCAACAGTGGG
