# Supplementary material for: Angiopoietin-2–integrin α5β1 signaling enhances vascular fatty acid transport and prevents ectopic lipid-induced insulin resistance
Source: Nat Commun. 2020 Jun 12;11:2980. doi: 10.1038/s41467-020-16795-4 (PMC7293240; doi:10.1038/s41467-020-16795-4)
Supplement: Supplementary file 3 — Reporting Summary [file 41467_2020_16795_MOESM3_ESM.pdf]

nature research

Corresponding author(s): Gou Young Koh

Last updated by author(s): Apr 17, 2020

Reporting Summary

Nature Research wishes to improve the reproducibility of the work that we publish. This form provides structure for consistency and transparency in reporting. For further information on Nature Research policies, see Authors & Referees and the Editorial Policy Checklist.

Statistics

For all statistical analyses, confirm that the following items are present in the figure legend, table legend, main text, or Methods section.

n/a	Confirmed	
☐	☒	The exact sample size (*n*) for each experimental group/condition, given as a discrete number and unit of measurement
☐	☒	A statement on whether measurements were taken from distinct samples or whether the same sample was measured repeatedly
☐	☒	The statistical test(s) used AND whether they are one- or two-sided<br>*Only common tests should be described solely by name; describe more complex techniques in the Methods section.*
☐	☒	A description of all covariates tested
☐	☒	A description of any assumptions or corrections, such as tests of normality and adjustment for multiple comparisons
☐	☒	A full description of the statistical parameters including central tendency (e.g. means) or other basic estimates (e.g. regression coefficient) AND variation (e.g. standard deviation) or associated estimates of uncertainty (e.g. confidence intervals)
☐	☒	For null hypothesis testing, the test statistic (e.g. *F*, *t*, *r*) with confidence intervals, effect sizes, degrees of freedom and *P* value noted<br>*Give P values as exact values whenever suitable.*
☒	☐	For Bayesian analysis, information on the choice of priors and Markov chain Monte Carlo settings
☒	☐	For hierarchical and complex designs, identification of the appropriate level for tests and full reporting of outcomes
☒	☐	Estimates of effect sizes (e.g. Cohen's *d*, Pearson's *r*), indicating how they were calculated

Our web collection on statistics for biologists contains articles on many of the points above.

Software and code

Policy information about availability of computer code

Data collection	The following software were used for data collection:<br>Zen Blue 2.3 software (Carl Zeiss)<br>Zen Black 2.3 software (Carl Zeiss)<br>QuantStudio 3 (Applied Biosystems)<br>edgeR 3.2.2 (Bioconductor)<br>Bowtie2 version 2.1.0. (BIOPAC Systems Inc)<br>Genowiz™ version 4.0.5.6 (Ocimum Biosolutions)
Data analysis	The following software were used for data analysis:<br>Zen Blue 2.3 software (Carl Zeiss)<br>Zen Black 2.3 software (Carl Zeiss)<br>ImageJ (Fiji version) Software (NIH)<br>GraphPad Prism 7.0 (GraphPad Software)<br>PASW Statistics 18 (SPSS)<br>MeV tm4 (TIGR)

For manuscripts utilizing custom algorithms or software that are central to the research but not yet described in published literature, software must be made available to editors/reviewers. We strongly encourage code deposition in a community repository (e.g. GitHub). See the Nature Research guidelines for submitting code & software for further information.

Data

Policy information about availability of data

All manuscripts must include a data availability statement. This statement should provide the following information, where applicable:

- Accession codes, unique identifiers, or web links for publicly available datasets
- A list of figures that have associated raw data
- A description of any restrictions on data availability

The RNA-seq data are available in the European Bioinformatics Institute (EMBL-EMI's) ArrayExpress under the accession number E-MTAB-6161. Other transcriptomic datasets analyzed in this study can be retrieved from the GEO repository under the accessions GSE20950, GSE29226, GSE29231, GSE16415, GSE71416 for the comparison of NDO vs DO, GSE80654 for human SVF and adipocytes, GSE2508 for NDO vs lean human adipocytes, GSE55200 for NDO vs lean human SAT, and GSE20950 for NDO human SAT vs VAT datasets. The source data underlying all Figs and Supplementary Figures are provided as a Source Data file. A Reporting Summary for this article is available as a Supplementary Information file. All other data that support the findings of this study are available from the corresponding author upon reasonable request.

Field-specific reporting

Please select the one below that is the best fit for your research. If you are not sure, read the appropriate sections before making your selection.

☒ Life sciences ☐ Behavioural & social sciences ☐ Ecological, evolutionary & environmental sciences

For a reference copy of the document with all sections, see nature.com/documents/nr-reporting-summary-flat.pdf

Life sciences study design

All studies must disclose on these points even when the disclosure is negative.

Sample size	Sample sizes were chosen on the basis of standard power calculations (with α = 0.05 and power of 0.8) performed for similar experiments and statistical methods were not used to predetermine sample sizes as previously published (Robciuc et al., Cell Metabolism, 2016; Shimizu et al., Journal of Clinical Investigation, 2014).
Data exclusions	No samples were excluded from the analysis.
Replication	Experiments were replicated at least once for all analyses and number of reproductions of each experimental finding is described in each figure legend. All attempts at experimental replication were successful.
Randomization	Animals from different cages, but within the same experimental group, were selected to assure randomization. Experiments involving in vitro study was assured randomization through double-blind experiments.
Blinding	The investigators were not blinded during experiments involving long term high fat diet challenge due to clear appearance of body mass changes among the groups. However, two independent investigators have performed most of experiments in parallel and administration of chemicals was carried out as blinded experiments. For other in vivo and in vitro studies, investigators were blinded during the experiments and quantifications. For RNA sequencing, data was analyzed in unsupervised manner.

Reporting for specific materials, systems and methods

We require information from authors about some types of materials, experimental systems and methods used in many studies. Here, indicate whether each material, system or method listed is relevant to your study. If you are not sure if a list item applies to your research, read the appropriate section before selecting a response.

Materials & experimental systems

n/a	Involved in the study	
☐	☒	Antibodies
☐	☒	Eukaryotic cell lines
☒	☐	Palaeontology
☐	☒	Animals and other organisms
☐	☒	Human research participants
☒	☐	Clinical data

Methods

n/a	Involved in the study	
☒	☐	ChIP-seq
☒	☐	Flow cytometry
☒	☐	MRI-based neuroimaging

Antibodies

Antibodies used	Primary antibodies (diluted at a ratio of 1:200 in blocking solution): anti-Perilipin (guinea pig polyclonal, 20R-PP004, Fitzgerald), anti-mouse CD31 (hamster monoclonal, 2H8, Millipore), anti-GFP (rabit polyclonal, AB3080, Millipore), anti-cleaved caspase 3 (rabbit polyclonal, 9661, Cell Signaling), anti-UCP1 (rabit polycloncal, ab23841, Abcam), anti-Integrin α5β1 (rat monoclonal,

BMB5, Millipore), anti- active Integrin β1 (rat monoclonal, 9EG7, BD Biosciences), anti- Integrin β1 (mouse monoclonal, 12G10, Abcam), anti-HA (rabbit polyclonal, H6908, Sigma Aldrich), and anti-human CD31 (rabit polyclonal, ab28364, Abcam).
Secondary antibodies (diluted at a ratio of 1:1000; all from Jackson ImmunoResearch): Cy3-conjugated anti-guinea pig antibody, Cy5-conjugated anti-hamster antibody, FITC-conjugated anti-rabbit antibody, FITC-conjugated anti-rat antibody, and FITC-conjugated goat antibody.

Validation	All the antibodies were validated for the species and applications (immunohistochemistry) by the correspondent manufacturer, which is described in the manufacturer's website. Our usage was described in the Methods section of the manuscript accordingly.<br>For both whole-mount and section staining, samples were permeabilized and blocked with blocking buffer containing 5% donkey (or goat) serum in 0.3% Triton-X 100 in PBS for 1 h at room temperature (RT). Samples were incubated with the primary antibody diluted in the blocking buffer overnight at 4 °C. After several washes with PBS, samples were incubated for 2 h at RT with the fluorocrome-conjugated secondary antibodies diluted in the blocking buffer. After several washes with PBS, samples were mounted with Vectashield (Vector Laboratories).

Eukaryotic cell lines

Policy information about cell lines

Cell line source(s)	HUVEC (ATCC), MS1 (ATCC), Human primary subcutaneous pre-adipocytes (ATCC), Mouse primary subcutaneous pre-adipocytes (primary culture), Mouse primary subcutaneous and visceral endothelial cells (primary culture).
Authentication	Cells were authentificated based on their morphology, growth condition and specific gene expression.
Mycoplasma contamination	Cell lines were tested negative for mycoplasma contamination.
Commonly misidentified lines (See ICLAC register)	No commonly misidentified cell lines were used in the study.

Animals and other organisms

Policy information about studies involving animals; ARRIVE guidelines recommended for reporting animal research

Laboratory animals	Specific pathogen–free C57BL/6J, Adiponectin-Cre, Integrinβ1 flox/flox, Tie2-GFP and RiboTag mice were purchased from Jackson Laboratory (Jackson Labs, Bar Harbor, ME). Angpt2-eGFP (Tg [Angpt2-EGFP] DJ90Gsat/Mmcd) were purchased from the Mutant Mouse Regional Resource Centers (MMRRC), Angpt2 flox/flox, Angpt2-lacZ, Adiponectin-Cre-ERT2, and VE-Cadherin-Cre-ERT2 mice were generous gifts from collaborators.<br>For all experiments, male mice aged 8-weeks-old under normal chow or short term high fat diet, and 16-weeks-old under long term high fat diet were used.<br>Mice were housed under 12 light/12 dark cycle, temperatures of 22±2°C with 50±10% humidity.
Wild animals	The study did not involve wild animals.
Field-collected samples	The study did not involve samples collected from the field.
Ethics oversight	Animal care and experimental procedures were performed under the approval from the Institutional Animal Care and Use Committee (No. KA2013-39) of KAIST.

Note that full information on the approval of the study protocol must also be provided in the manuscript.

Human research participants

Policy information about studies involving human research participants

Population characteristics	Human subcutaneous adipose tissues were collected from female patients (ages 39-56) undergoing breast reconstruction after mastectomy for breast cancer.
Recruitment	All human samples were collected in an unbiased manner by the tissue bank of Seoul National University Hospital, Seoul, Korea, with the informed consents from the donors following the bioethics and safety regulations.
Ethics oversight	The Institutional Review Board of Seoul National University Hospital (1708-043-876) approved experimental procedures with human adipose tissue specimens.

Note that full information on the approval of the study protocol must also be provided in the manuscript.
